# An online monitoring method of milling cutter wear condition driven by digital twin

**DOI:** 10.1038/s41598-024-55551-2

**Published:** 2024-02-29

**Authors:** Xintian Zi, Shangshang Gao, Yang Xie

**Affiliations:** 1 School of Mechanical Engineering, Jiangsu University of Science and Technology, Zhenjiang, 212000 China; 2 Taian Haina Spindle Science & Technology Co., Ltd, Taian, 271000 China

**Keywords:** Tool wear, Ensemble learning, Digital twin, Feature engineering, Electrical and electronic engineering, Mechanical engineering

## Abstract

Real-time online tracking of tool wear is an indispensable element in automated machining, and tool wear directly impacts the processing quality of workpieces and overall productivity. For the milling tool wear state is difficult to real-time visualization monitoring and individual tool wear prediction model deviation is large and is not stable and so on, a digital twin-driven ensemble learning milling tool wear online monitoring novel method is proposed in this paper. Firstly, a digital twin-based milling tool wear monitoring system is built and the system model structure is clarified. Secondly, through the digital twin (DT) data multi-level processing system to optimize the signal characteristic data, combined with the ensemble learning model to predict the milling cutter wear status and wear values in real-time, the two will be verified with each other to enhance the prediction accuracy of the system. Finally, taking the milling wear experiment as an application case, the outcomes display that the predictive precision of the monitoring method is more than 96% and the prediction time is below 0.1 s, which verifies the effectiveness of the presented method, and provides a novel idea and a new approach for real-time on-line tracking of milling cutter wear in intelligent manufacturing process.

## Introduction

The CNC milling machine is the main equipment for the automated machining process, milling cutter is an important basic component of the CNC milling machine. The tool is in direct touch with the workpiece in machining and acts as the main executor of the milling process^1,2^. Tool wear is unavoidable when processing workpieces, serious tool wear will extend the workpiece processing cycle, and reduce workpiece processing accuracy and surface quality, thus affecting the production cost and processing efficiency. According to statistics, tool failure maintenance costs are 15 to 40% of overall manufacturing costs^3^. The stoppage time resulting from tool failure takes up approximately 20% of the overall stoppage time of the tool^4^. However, the current degree of automation of online tool wear monitoring is relatively low, relying largely on the experience of skilled workers to determine tool wear^5,6^. The DT is an example of a DT that is a combination of the physical space and the physical space. DT as the main method of real-time mapping between physical space and twin space^7^, opens up a new way for realizing tool wear state monitoring on CNC machine tools. Therefore, there is an urgent need for a digital twin-cutting tool wear state monitoring and provide real-time online autonomous tool change decision-making methods in the machining process.

Domestic and foreign scholars have carried out considerable research on tool wear monitoring. Awasthi et al.^8^ utilized sensor measurements of power and force for predicting deviations in power output and cutting forces during normal operation, combined with a model-based fault detection and isolation approach to determine the optimal sensor suite and test setup for improving tool wear monitoring performance during milling. The indirect measurement method has been commonly adopted in the field of tool wear status monitoring owing to its advantages such as easier and more flexible installation, lower monitoring cost, and the ability to detect online tool wear^9,10^. With the development of machine learning technology, many scholars have constructed various predictive models for tool wear monitoring. He et al.^11^ used sensors to collect vibration, force, and acoustic emission signals during tool cutting to obtain time series data of the tool wear process and combined with the long and short-term memory convolutional neural network (LSTM-CNN) prediction model to accurately predict the tool wear. Bernini et al.^12^ proposed an unsupervised milling cutter wear monitoring method under variable process parameters and lubrication conditions, combined with different experimental data to verify the effectiveness of the proposed method and improve the accuracy of milling cutter wear prediction. Yan et al.^13^ proposed a highly condition-adaptive method for tool wear monitoring under time-varying operating conditions, and the results show that the method can reliably assess the wear state and remaining service life of the tool. Sun et al.^14^ utilized long and short-term memory network prediction and residual convolutional neural networks combined with raw signals acquired during machining to achieve tool state monitoring. Kong et al.^15^ used the feature downscaling technique to obtain features that can better characterize the tool wear state, combined with gaussian process regression (GPR) to achieve tool wear state monitoring. Nie et al.^16^ proposed an improved particle swarm optimized (IPSO) least squares support vector machine (LS-SVM) prediction model for tool wear status monitoring. However, the above literature often uses a single prediction model for tool wear prediction, which makes it easy to ignore the indispensable information in the tool wear process and the results have a certain degree of chance.

To address the issue of single-model prediction, some experts have adopted ensemble algorithms to study tool wear. The ensemble algorithm, as a branch of machine learning, relies on multiple base learners and corresponding weights to comprehensively extract tool wear process information and improve the stability of model prediction. Nasir et al.^17^ used multi-sensor signals, combined with random forest (RF) and extreme gradient boosting (XGBoost) algorithm to predict tool wear status. Yuan et al.^18^ utilized the variational modal decomposition algorithm for signal processing, combined with an ensemble algorithm to establish a classification prediction model to enhance the accuracy of the tool wear prediction model. Li et al.^19^ adopted the gradient boosting decision tree (GBDT) to choose the best subset of features for tool wear status identification and combined GBDT with hybrid classification RBM (H- ClassRBM) to construct a prediction model. The experimental findings reveal that the ensemble model has better identification accuracy and stability compared to an individual model. Although the ensemble algorithm can enhance the accuracy and stability of tool wear prediction, the algorithm is based on offline historical data and ignores the influence of uncertainty factors such as the internal of CNC machine tools and equipment on tool wear, which leads to the inevitable bias of the prediction model.

In recent years, the DT has received great attention from scholars, and domestic and foreign scholars have been actively exploring the application of DT technology in the visualization and monitoring of intelligent workshops, real-time fault diagnosis of equipment, and full life cycle control of products^20,21^. DT technology with a high degree of physical information fusion and virtual reality synchronization is capable of depicting and modeling the features, actions, and performances of physical spatial objects during the whole process of lifecycle^22,23^. Guo et al.^24^ utilize the improved GJK collision detection algorithm to detect the collision model of the tool and the machine tool, combined with the collection of tool wear data, to realize the online prediction of tool wear and reduce the risk of tool collision. Liu et al.^25^ presented an online real-time monitoring method for milling cutter wear status based on the DT and clarified the five-layer system architecture of the DT. Zhang et al.^26^ used DT technology, combined with tool wear monitoring in the cutting process, to realize real-time online management and control of tool wear. Zhang et al.^27^ utilized a transfer learning strategy and combined DT technology to construct a tool wear monitoring model for varying operating conditions. However, the above scholars only focus on the application of digital twins in tool wear and do not study the combination of multi-model tool wear prediction methods and digital twins.

In the actual cutting process, there are individual tool wear prediction models with errors and unstable results, as well as the DT model based on historical data makes it difficult to realize real-time tool wear monitoring. Therefore, this paper presented a digital twin-driven ensemble learning milling tool with wear online monitoring method. On the one hand, DT technology is used to address the problem that real-time online monitoring cannot be realized based on offline historical data, and on the other hand, the accuracy and stability of model prediction can be improved by using ensemble learning.

## Digital twin-driven ensemble learning framework for online monitoring methods of milling tools

### System architecture

**Figure 1:**
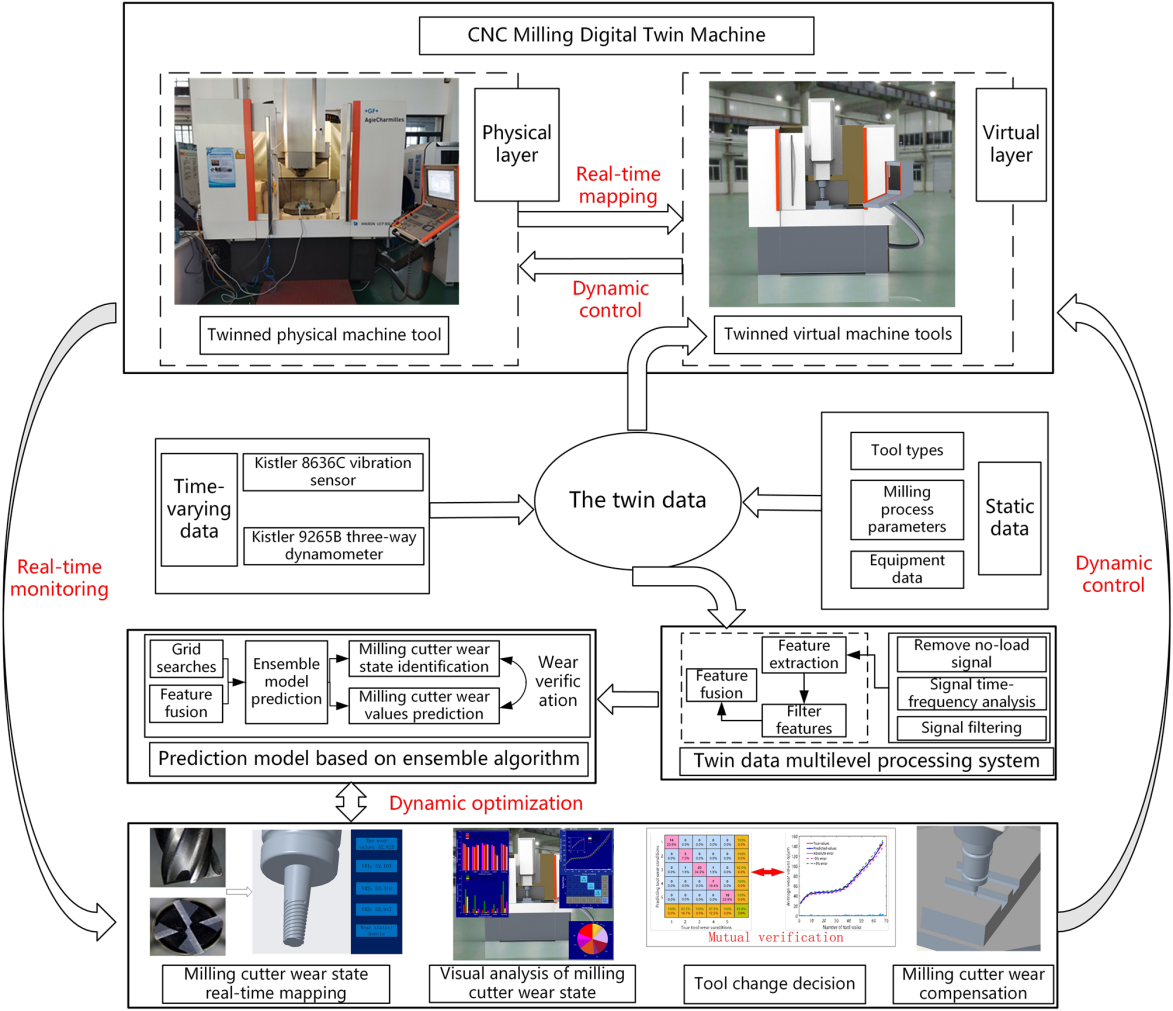
A methodological framework for online monitoring of DT milling cutter wear.

The digital twist-driven ensemble learning milling tool wear online monitoring method introduced in this paper is displayed in Fig. 1. Firstly, the twin virtual machine tool of the physical machine tool is established, combining different sensors to collect real-time data and the attribute data of the CNC machine tool itself to get the twin data of the CNC machine tool. Then, the twin data processing system is established by using data processing technology. The milling tool wear prediction model is constructed by the ensemble algorithm, and the milling tool wear is mutually verified by combining the model prediction results. Finally, based on the DT and ensemble algorithms to build the milling cutter wear state real-time mapping, milling cutter wear status real-time visualization and analysis, as well as tool change decision-making and compensation, and dynamic control of the physical CNC machine tool real-time parameter adjustment and tool wear optimization, to achieve the real-time dynamic monitoring of milling cutter wear in the actual cutting process of the CNC machine tool.

1. Twin data acquisition: real-time data acquisition is the basis for the operation of the digital twin system. Static data such as machining process parameters, equipment information, and tool types are acquired through the manufacturing equipment system (MES). The coordinates of tools and workpieces are acquired through open interfaces to the machine's numerical control system. The dynamic data such as vibration and cutting force are acquired by using external sensors. The data in the cutting process of the machine tool is collected in real-time and uploaded to the digital twin system.
2. Tool wear model prediction: using the XGBoost ensemble model to predict the tool wear value and wear state online is the core. The dynamic data of the machine tool is collected in real-time, and then the data is input into the digital twin multi-level data processing system for data processing and feature downscaling, and the processed features are input into the XGBoost ensemble model of tool wear to predict the tool wear value and wear state online.
3. Real-time online tool wears monitoring and mutual verification of tool wear: the tool wear curve is drawn online through the tool wear value, and the tool compensation strategy is formulated online in real time. The tool wear status is utilized to identify tool wear online and develop tool change decisions. At the same time, the predicted tool wear state and the predicted tool wear value are mutually verified to improve the accuracy of digital twin tool wear monitoring.

### DT system development and configuration

In this paper, Creo 6.0, Unity 3D 2021, and 3ds Max 2017 software are used to develop a DT system for detecting the wear status of milling tools on CNC machine tools. Firstly, Creo 6.0 is used to model the Mikron UCP 800 5-axis machining center in 3D according to the size scale. Then the model is imported into 3ds Max 2017 software to create cutting and machining process animation as well as rendering the CNC machine model. Finally, Unity 3D 2021 is employed to design the system interface and program interaction. The data from the actual CNC machine is fed to the tool wear DT through TCP/IP communication protocol.

### Methodology study for modeling DT mechanisms

The geometric model and physical model support the tool behavior model^5^, and the behavior model is the reference of the rule model^28^, so the twin mechanism model reflecting the characteristics of the actual tool is constructed by an ensemble of the four sub-models.

#### Geometric models

The tool geometric model mainly includes parameters such as tool rake angle and tip radius^29^. From Eq. (1), it can be seen that the tool wear rate is mainly affected by the front and back angles of the tool.

1 $$\frac{{dV_{B} }}{dt} = \frac{dW}{{dt}} \cdot \frac{{\cot \alpha_{n} - \tan \gamma_{n} }}{{\omega V_{B} }}$$

where $\frac{{dV_{B} }}{dt}$ is the tool wear rate; $W$ is the wear volume; $\alpha_{n}$ is the front Angle of the tool; $\gamma_{{\text{n}}}$ is the back Angle of the tool; $\omega$ is the angular velocity of the tool rotation.

#### Physical models

The physical model mainly reflects the material attributes of the tool and the workpiece, and the construction of the physical model comprehensively reflects the macroscopic properties of the material, mainly the relationship among the parameters of the material, such as stress, strain, temperature, etc^30^. The Johnson–Cook model is used, and its expression is as follows:

2 $$\overline{\sigma } = \left[ {A + B\left( {\overline{\varepsilon }^{pl} } \right)^{n} } \right]\left[ {1 + C\ln \left( {\frac{{\mathop {\overline{\sigma }}\limits^{\,\,\,\,\,\,\,\,. \,\,\,\, pl} }}{{\mathop {\overline{\sigma }}\limits^{.} }}} \right)} \right]\left[ {1 - \left( {\frac{{T - T_{{\text{g}}} }}{{T_{m} - T_{g} }}} \right)^{m} } \right]$$

where $A$ is the yield strength of the material; $B$ is the hardening modulus of the material; $C,m,{\text{n}}$ is the material coefficient; $\overline{\varepsilon }^{pl}$ is the material equivalent plastic strain; $\mathop {\overline{\varepsilon } }\limits^{.}$ is the material reference strain rate; $\mathop {\overline{\varepsilon } }\limits^{\,\,\,\,\,\,\,\,. \,\,\,\, pl}$ is the material equivalent plastic strain rate; $T_{m}$ is the melting point of the material; $T_{g}$ is the ambient temperature.

#### Behavioral models

The friction generated by tool and chip interaction seriously affects the quality of the workpiece's appearance^31^. A hybrid friction model is adopted to represent the contact friction situation with the following equation:

3 $$\tau_{f} = \left\{ {\begin{array}{*{20}c} {\mu a_{k} ,\tau_{f} < \tau_{\max } } \\ {\tau_{\max } ,\mu a_{k} > \tau_{\max } } \\ \end{array} } \right.$$

where $\mu$ is the friction coefficient; $a_{k}$ is the contact normal stress.

The chip separation criterion and fracture criterion are established based on the separation criterion and rupture stress criterion of equivalent plastic strain. In this paper, the J-C fracture criterion is selected as shown in Eq. (4).

4 $$\varepsilon_{p}^{f} = \left[ {d_{a} + d_{b} \exp \left( {\frac{\sigma }{{\sigma_{e} }}} \right)} \right]\left[ {1 + d_{c} \ln \left( {\frac{{\mathop {\sigma_{p} }\limits^{.} }}{{\mathop {\sigma_{n} }\limits^{.} }}} \right)} \right]\left[ {1 + d_{d} \left( {\frac{{T - T_{g} }}{{T_{m} - T_{g} }}} \right)} \right]$$

where $\varepsilon_{p}^{f}$ is cumulative damage; $d_{a} ,d_{b} ,d_{c} ,d_{d}$ are the material coefficient; $\sigma$ is the material tensile stress; $\sigma_{e}$ is the material equivalent stress; $\mathop {\sigma_{n} }\limits^{.}$ is the corresponding plastic stretch; $\mathop {\sigma_{p} }\limits^{.}$ is the corresponding plastic stretch when the element fails; $T$ is the deformation temperature; $T_{m}$ is the melting point of the material; $T_{g}$ is room temperature.

#### Rule models

To reflect the milling cutter wear degradation principle, this paper adopts the rule modeling based on the milling cutter wear mechanism. The material of the milling cutter is a carbide three-tooth face milling cutter, and the workpiece material is a TC4 titanium alloy plate. The wear mechanism to get the milling cutter wear corresponds to the degradation model^32^, as shown in Eq. (5).

5 $$\frac{dW}{{dt}} = M\left( {\nu ,f} \right) + Qexp\left( {\frac{ - g}{{RT}}} \right)$$

where $M,Q$ are cutting constants; $f$ is the amount of tool feed; $\nu$ is the cutting speed of tool; $R$ is the temperature constant; $T$ is the universal gas constant; $g$ is the cutting activation energy constant.

## Digital twin-driven multi-level data processing of milling cutter wear

**Figure 2:**
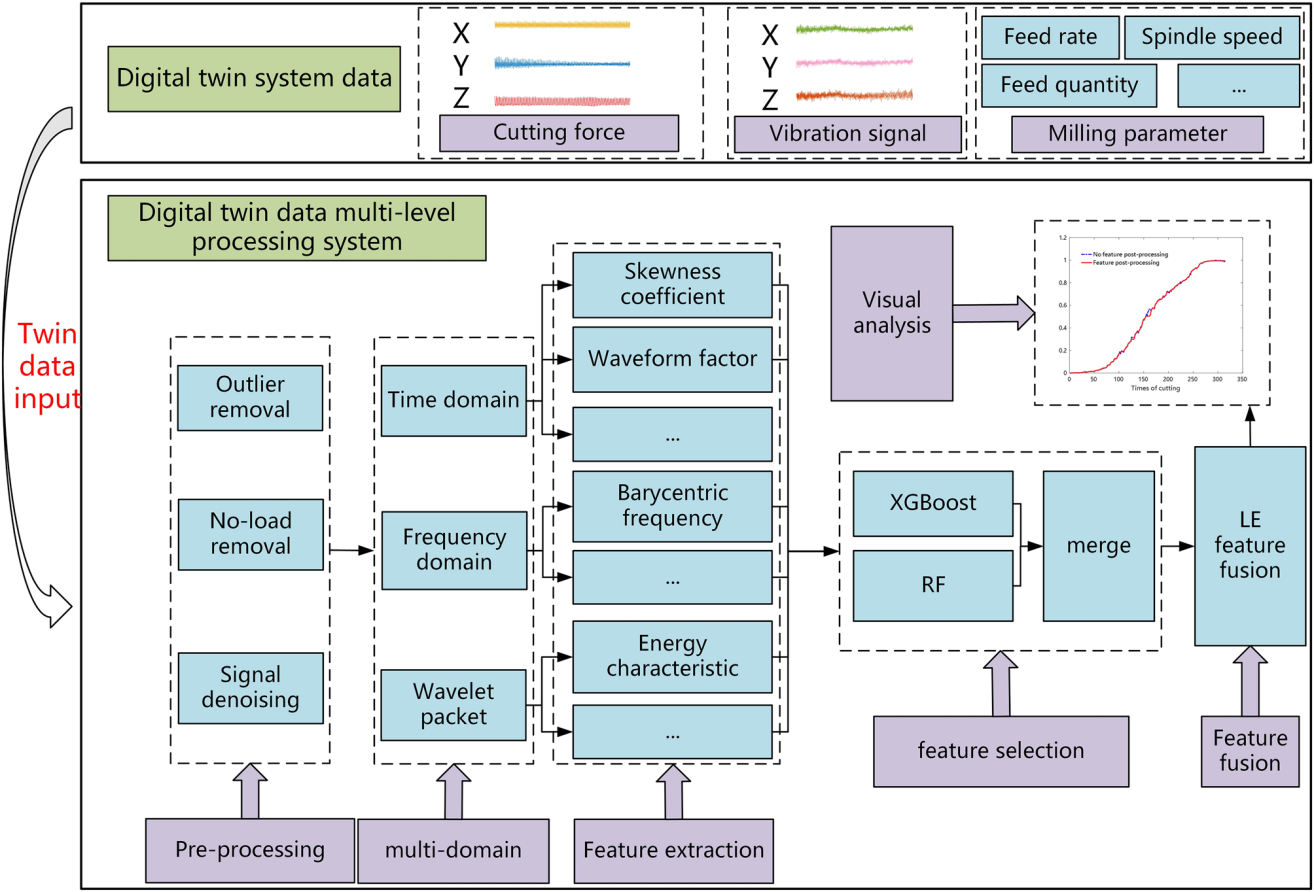
Multi-level data processing system for DT milling cutter wear.

The DT data processing system is used to remove interfering information and improve the characterization of twin data. Figure 2 displays the multi-level data processing of the DT system for milling cutter wear.

### Twin data pre-processing

**Figure 3:**
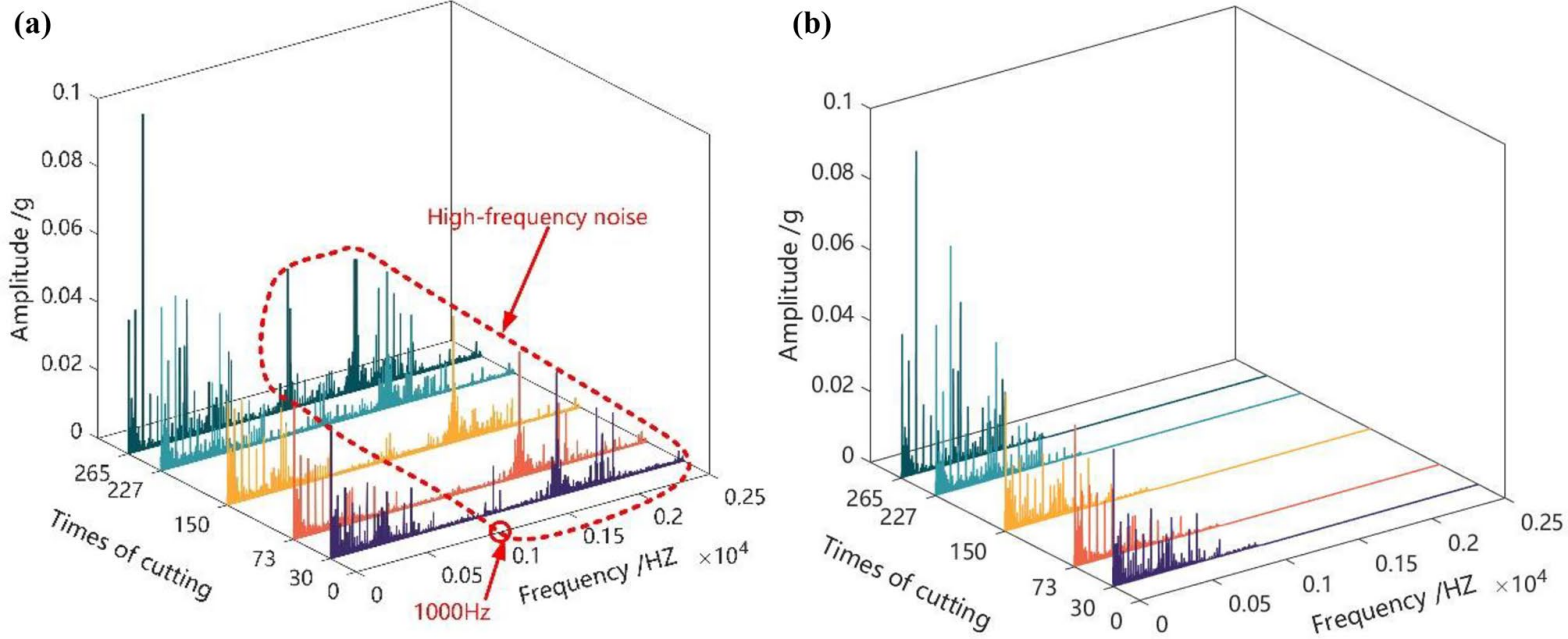
A three-way vibration signal to remove noise. (**a**) Frequency distribution of vibration signal noise (**b**) Frequency distribution of vibration signal after denoising.

Due to the periodic cutting in and out of the tool teeth during milling, the cutting vibration signal also shows periodicity, so the low-frequency part of the acquired signal is mainly the effective vibration signal, while the high-frequency part is the interference noise signal^33^. Fourier transform is employed to convert the time-domain signal into the frequency-domain signal, which is convenient to observe the frequency information of the signal^34^. A low-pass filter is used for filtering, and the noise signal is concentrated above 1000 HZ, so the window function low-pass filter is selected, the window function is $Balckman$ window, and the cutoff frequency is 1000 Hz, as shown in Fig. 3a. Figure 3b shows the spectrum of the X-direction vibration signal after denoising. After data preprocessing, the twin data information is obtained that better mirrors the tool wear course.

### Twin data feature selection and feature fusion

The time-domain, frequency-domain, and time–frequency-domain features of the vibration signals and cutting force signals are extracted, of which the time-domain features are 8, the frequency-domain features are 4, and the time–frequency-domain features are 16. At the same time, each signal corresponds to the X, Y, and Z directions, and a total of (8 + 4 + 16)$\times$ 2 $\times$ 3 = 168 features are extracted. These 168 features constitute a 168-dimensional features vector reflecting the tool wear.

**Figure 4:**
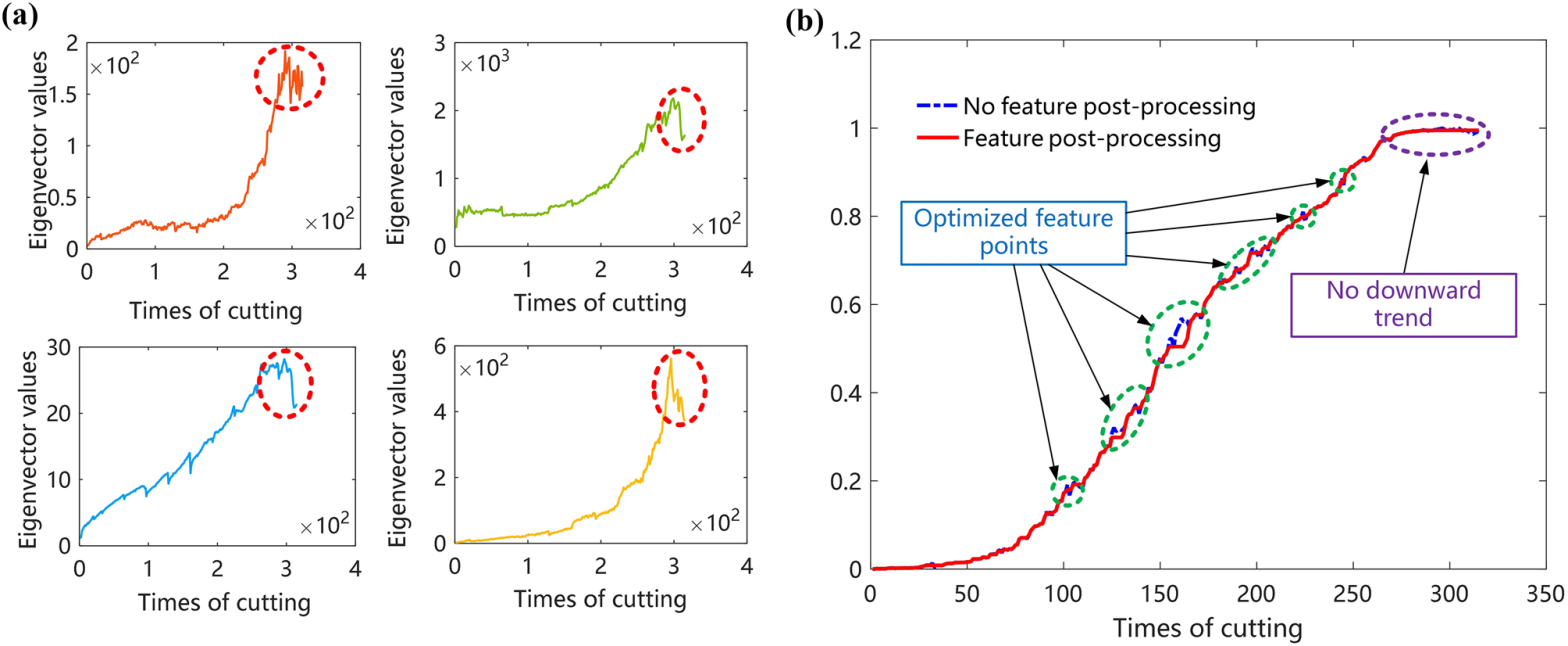
Features results. (**a**) Features selection results. (**b**) Features fusion and features post-processing.

Ensemble learning feature selection algorithms all use their algorithms themselves to rank the importance of features, and different algorithms have different indicators of the importance of the signal. Therefore, RF and XGBoost algorithms are employed for multi-algorithm feature selection. Randomly selected four features in the above selection of features for plotting, as shown in Fig. 4a, the screening features basically maintain an upward trend and there is a powerful positive association with the actual tool wear curve. However, as can be seen from Fig. 4, the features after the feature selection show different degrees of decreasing trends (marked with red dashed lines in Fig. 4a).

For the problem of decreasing trend of features in feature selection, it is necessary to further optimize the features for downscaling. By first feature selection and then feature fusion multi-level feature downscaling, the importance of feature information can be effectively avoided from being lost. Finally, the original feature matrix is reduced to one dimension, and the compressed feature fusion downscaling results in between (0, 1). Although the twin data features after LE downgrading conform to the tool wear process as a whole, there are still small local fluctuations. The feature vectors after downscaling are optimized by feature post-processing to improve the characterization effect of the features on tool wear. Exponential smoothing and order-preserving regression are carried out for feature post-processing. Exponential smoothing eliminates localized sudden rises and falls, making the trend of the curve smoother. The combination of order-preserving regression can ensure that the curve is monotonically non-decreasing. As shown in Fig. 4b, the curve after feature post-processing is smoother and shows an overall upward trend, and effectively alleviates the trend of feature decline, which can better reflect the actual process of tool wear.

## Online prediction model of milling cutter wear for digital twins based on ensemble learning

An ensemble learning-based DT milling cutter wear prediction method is proposed. First, the sensor acquisition constructs twin data, and the ensemble learning model input is obtained using the twin data multi-level processing system. Then, the parameters of the XGBoost ensemble learning model are optimized by the grid search (GS) algorithm. Finally, the milling cutter wear value and wear status are predicted from both regression and classification of the ensemble prediction model, and the milling cutter wear is mutually verified based on the prediction results to enhance the milling cutter wear status recognition accuracy further.

### Optimization of prediction model parameters based on GS

**Figure 5:**
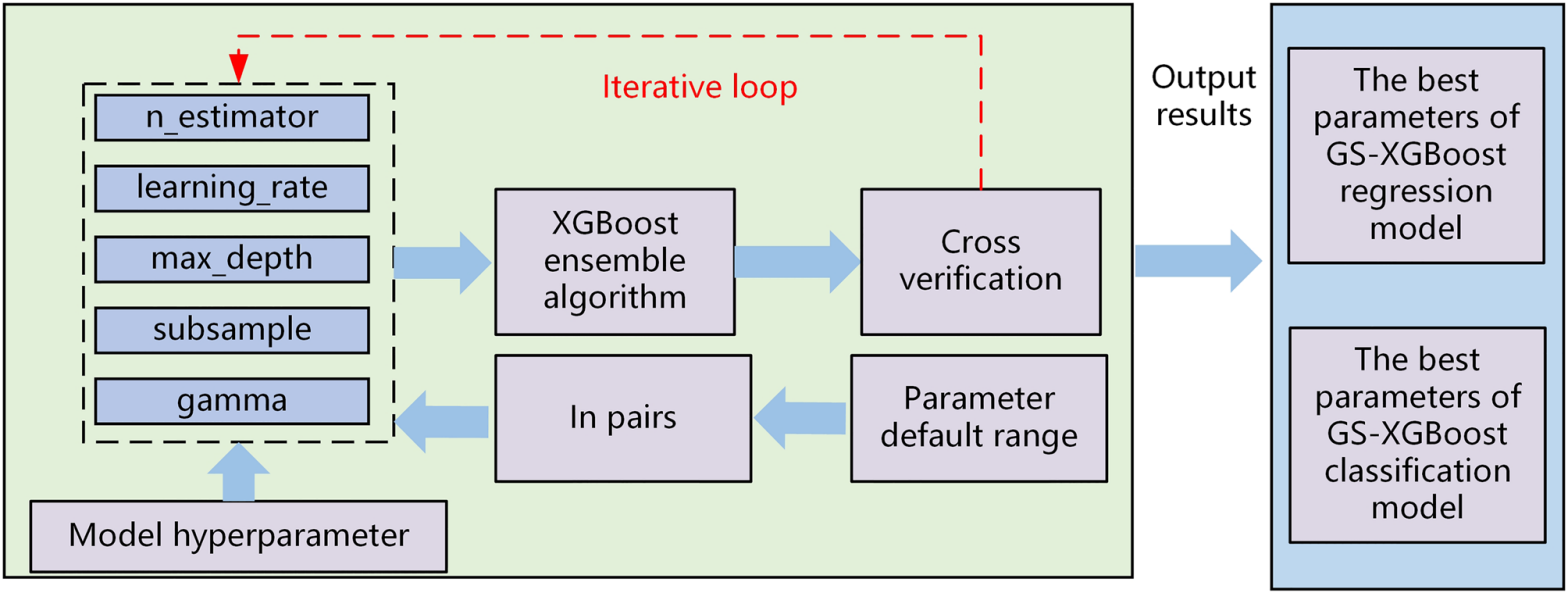
Optimization of XGBoost integration model parameters based on GS.

XGBoost model hyperparameters are more, but only part of the hyperparameters play a role in determining the model, and the rest of the hyperparameters take the default value. The impact of XGBoost hyperparameters on the model and the optimal range of values are combined to determine the hyperparameters that need to be optimized. Considering that GS is an exhaustive optimization algorithm, with the increase of hyperparameters, the model computation grows explosively. To reduce the amount of modeling calculations, this paper adopts a two-by-two combination of hyperparameters, combined with the iteration step of each hyperparameter, to determine the specific values of model parameters. The detailed process is described in Fig. 5.

### The milling cutter wears an online prediction model

**Figure 6:**
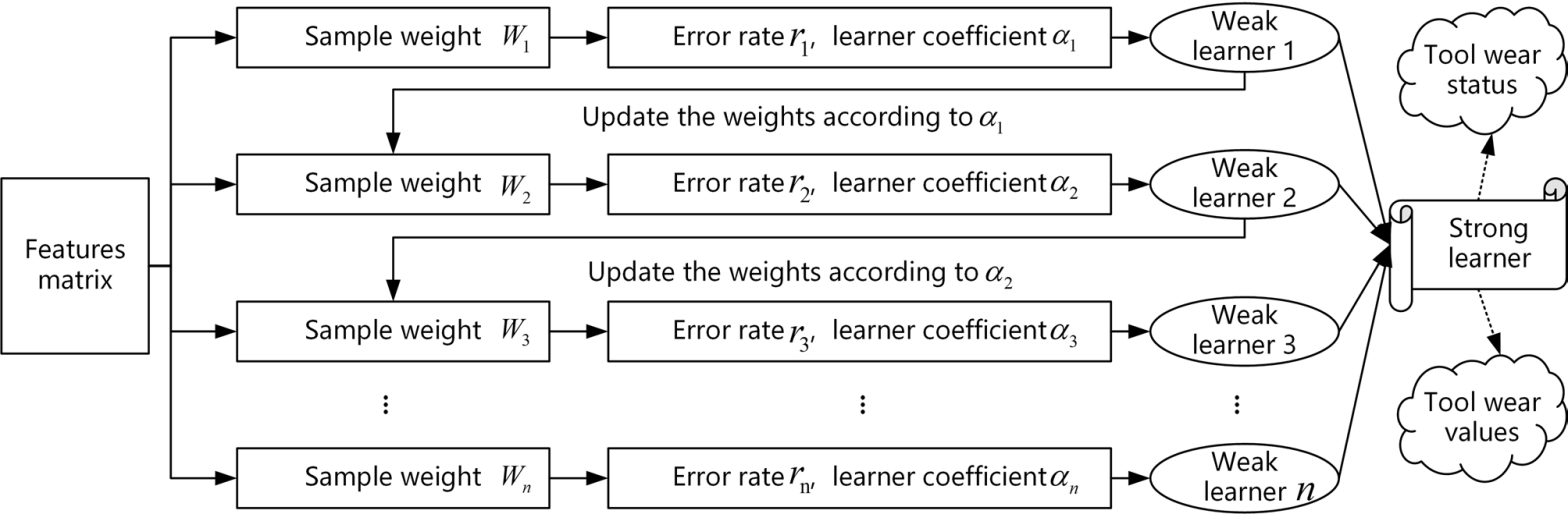
Principle of ensemble learning.

In this paper, the XGBoost ensemble learning model is based on updating the weights to obtain a strong learner, which in turn predicts the milling cutter wear status and milling cutter wear values. The ensemble model prediction principle is illustrated in Fig. 6.

Compared to the conventional ensemble algorithm, the main advantages of the XGBoost algorithm are listed below:

First, the algorithm is based on the GBDT algorithm model to optimize the loss function, increase the base classifiers, and add regularization terms, which makes the XGBoost algorithm more accurate in prediction, more flexible, and effective in preventing the model from overfitting.

Secondly, the XGBoost algorithm supports multi-threaded parallel computing and self-pruning, which can effectively improve the speed of model prediction and prevent the model from falling into local optimal solutions^35^.

## The case study

### Introduction to the experiment

**Figure 7:**
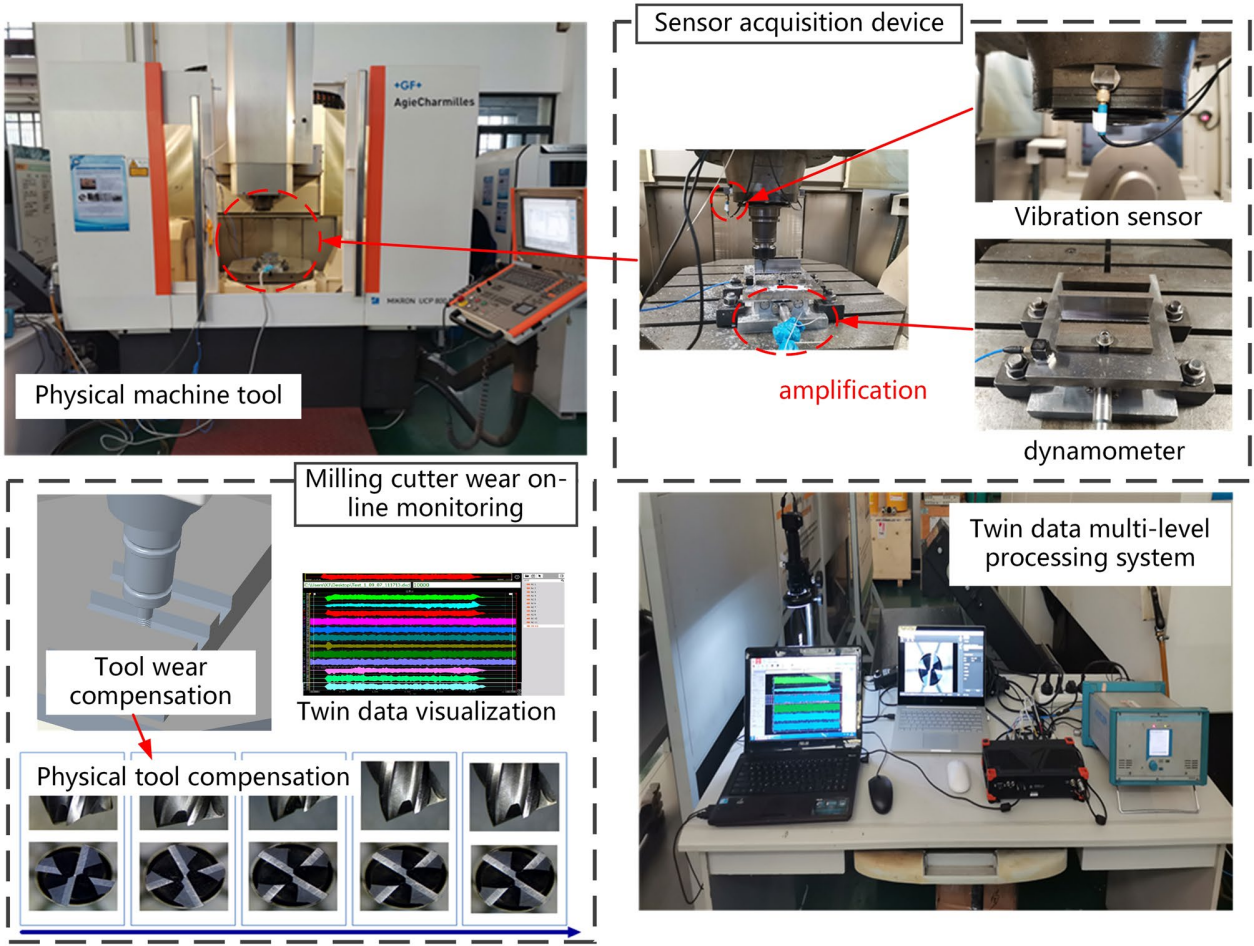
Diagram of experimental setup.

In this paper, the milling wear experiment was implemented on a Mikron UCP 800 5-axis machining center in a temperature-controlled workshop at 25 °C. Two types of sensors were employed to track the wear of the milling cutter. The Kistler 8636C vibration acceleration sensor was mounted on the workpiece. The Kistler 9265B three-way force gauge was mounted between the table and the workpiece. The experimental setup and its mounting position are shown in Fig. 7.

The workpiece is a TC4 titanium alloy plate with a size specification of 100 mm × 80 mm × 16 mm. The milling tool is a coated carbide three-tooth face milling cutter with a diameter of 8mm. The signals collected from different sensors are fed into the DEWEsoft data acquisition system, and the data are fed into the DT milling cutter wear monitoring system. After each tool run of the milling cutter, the wear value of the milling cutter is observed and recorded using an FM1.6 microscope to obtain the wear value of the whole process of tool wear and the tool wear status. Considering the use of a three-tooth face milling cutter in this experiment, three points are set on each tooth to measure the wear value, and the maximum wear value is used as the cutter's wear value $VB$. The twin milling cutter wear compensation strategy is utilized to compensate for the physical milling cutter wear in different wear statuses. A total of 270 tool walks were performed in this experiment, and the experimental equipment parameters and machining parameters are provided in Table 1.

**Table 1:** Experimental equipment and milling machining parameters.

Experimental equipment information		Processing parameter information	
Experimental equipment	Specific models	Parameter names	Parameter values
CNC machine tool	Mikron UCP 800 5-axis machining centers	Spindle speed $n$	1395 rpm
Dynamometer	Kistler 9265B 3-direction dynamometer	Radial depth of cut $a_{e}$	8 mm
Vibration sensor	Kistler8636C	Axial depth of cut $a_{p}$	0.3 mm
Wear values measuring device	FTM1.6 microscope	Cutting speed $V_{c}$	35 m/min
Data acquisition system	DEWEsoft	Feed speed $F$	670 mm/min
Milling material	TC4 titanium alloy plate	Number of effective teeth $z$	4(ϕ8)
Cutting tool material	Carbide three-tooth face milling cutter	Feed per tooth $f_{z}$	0.12 mm/tooth

### Construction of data sets

The CNC milling wear experiment to build the experimental data set, which includes the sensor acquisition of tool wear process characteristics signal, milling cutter wear value, and machining process parameters, specifically as depicted in Table 2. The milling tool wear status of this experiment was divided into five categories^36^, and the five categories were coded as 1, 2, 3, 4, and 5 in turn, with the five categories corresponding to the whole process of the tool from the beginning of milling to tool failure. Python language, jupyter notebook is deployed as a compiler with the model inputs as features of the twin data processing system. Two datasets are utilized in this paper, one is the PHM 2010 experimental dataset with a total sample size of 315 sets, and the other is the experimental dataset obtained from the experiments conducted in this paper with a total sample size of 270 sets. Considering the existence of tool wear on the time dimension correlation relationship and the training set and test set samples need to reflect the whole process of milling cutter wear, therefore, this paper adopts every interval of 3 samples to take 1 sample as the test set, and the final division of the training set and the test set of the ratio of 3:1.

**Table 2:** Data set details.

Data name	Data description	Sample frequency
Vibration signals	$A = \left\{ {A_{{\text{x}}} ,A_{y} ,A{}_{z}} \right\}$	5 ${\text{KHz}}$
Milling force signals	$F = \left\{ {F_{x} ,F_{y} ,F_{z} } \right\}$	
Wear values	$V = \left\{ {VB} \right\}$	1 piece of data per tool travel
Processing parameters	$D = \left\{ {n,a_{p} ,a_{e} ,V_{c} ,F,z,f_{z} } \right\}$	

This paper uses AdvantEdge finite element simulation simulation software to construct the twinning mechanism model of the tool and perform finite element simulation of the tool. The workpiece material is a TC4 titanium alloy plate (Ti-6Al-4V), the tool material is a cemented carbide three-tooth face milling cutter, and the parameter settings of the geometric model are as shown in Table 3. The physical model is selected as the Johnson–Cook intrinsic model, and the parameter settings of the physical model are shown in Table 4. The behavioral model adopts the traditional Cullen friction model, and the friction coefficient is $\mu = 0.2$. The J-C fracture criterion is selected for the chip separation and fracture criterion, and the behavioral parameters are set as shown in Table 5.

**Table 3:** Geometric model parameter settings.

Cutting tool diameter /mm	$\alpha_{n}$	$\gamma_{n}$	$\omega$
8	10	6	400

**Table 4:** Physical model parameter settings.

Workpiece material	$A\;({\text{MPa}})$	$B\;({\text{MPa}})$	$C$	$m$	$n$	$T_{m} \;({\text{K}})$	$T_{g} \;({\text{K)}}$
Ti-6Al-4V	752	489	0.016	0.024	1.02	1453	290

**Table 5:** Behavioral model parameter settings.

Workpiece material	$d_{a}$	$d_{b}$	$d{}_{c}$	$d_{d}$
Ti-6Al-4V	752	489	0.016	0.024

### Model prediction and analysis of results

Milling cutter wear values and wear status are important indicators of milling cutter wear condition. Milling cutter wear values prediction can directly predict the specific wear values of the milling cutter, provide a reference for milling cutter wear compensation, and improve the machining quality of the workpiece. Therefore, this paper establishes an ensemble prediction model from both the regression model and classification model, the regression ensemble prediction model can get the milling cutter wear value, and the classification ensemble prediction model can get the milling cutter wear state.

Multiple metrics were used to evaluate regression and classification models. The root mean square error (RMSE) and determination coefficient $R^{2}$ were used to evaluate the prediction accuracy of the regression model. Accuracy and f1 values are used to evaluate the classification accuracy of the model. At the same time, the model is compared from the time dimension, and the model prediction speed is judged by comparing the model prediction time. Ensemble models such as RF, GBDT, XGBoost, light gradient boosting machine (LGBM), and Stacking models are used to predict milling cutter wear from both regression and classification aspects, as shown in Table 6.

**Table 6:** Evaluation metrics of model prediction results.

Prediction models	Evaluation metrics of regression model			Evaluation metrics of classification model		
	RMSE	$R^{2}$	Prediction time	Accuracy	f1 values	Prediction time
RF	1.8659	0.9723	0.143651	0.8656	0.8508	0.28497
GBDT	3.0036	0.8612	0.067542	0.9254	0.9275	0.03147
XGBoost	1.4986	0.9831	0.045271	0.9701	0.9623	0.01457
LGBM	2.6487	0.9220	0.069831	0.5948	0.5882	0.02761
Stacking	1.7362	0.9664	0.321549	0.9104	0.9056	0.07968

**Figure 8:**
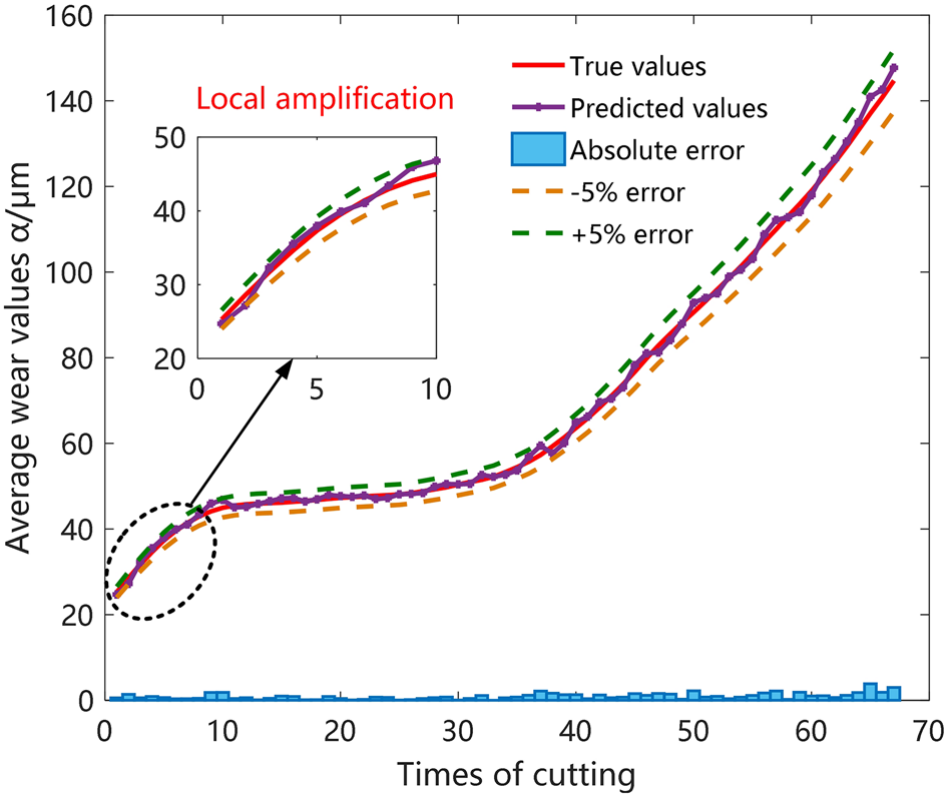
Milling cutter wear prediction curve.

In terms of model $R^{2}$, RF, XGBoost and Stacking models perform better with $R^{2}$ above 95%, and the XGBoost model has the highest $R^{2}$ and the smallest RMSE, which indicates the model with highly accurate prediction. From the perspective of model prediction time XGBoost, GBDT, and LGBM models perform better, and the XGBoost model takes the least time to predict and has the fastest prediction speed. Therefore, from the ensemble model forecast time and accuracy comprehensive analysis XGBoost model performs best. As reflected in Fig. 8, there is a small and smooth absolute error in the predicted and real wear curves, and the model prediction errors are all controlled within 5%.

**Figure 9:**
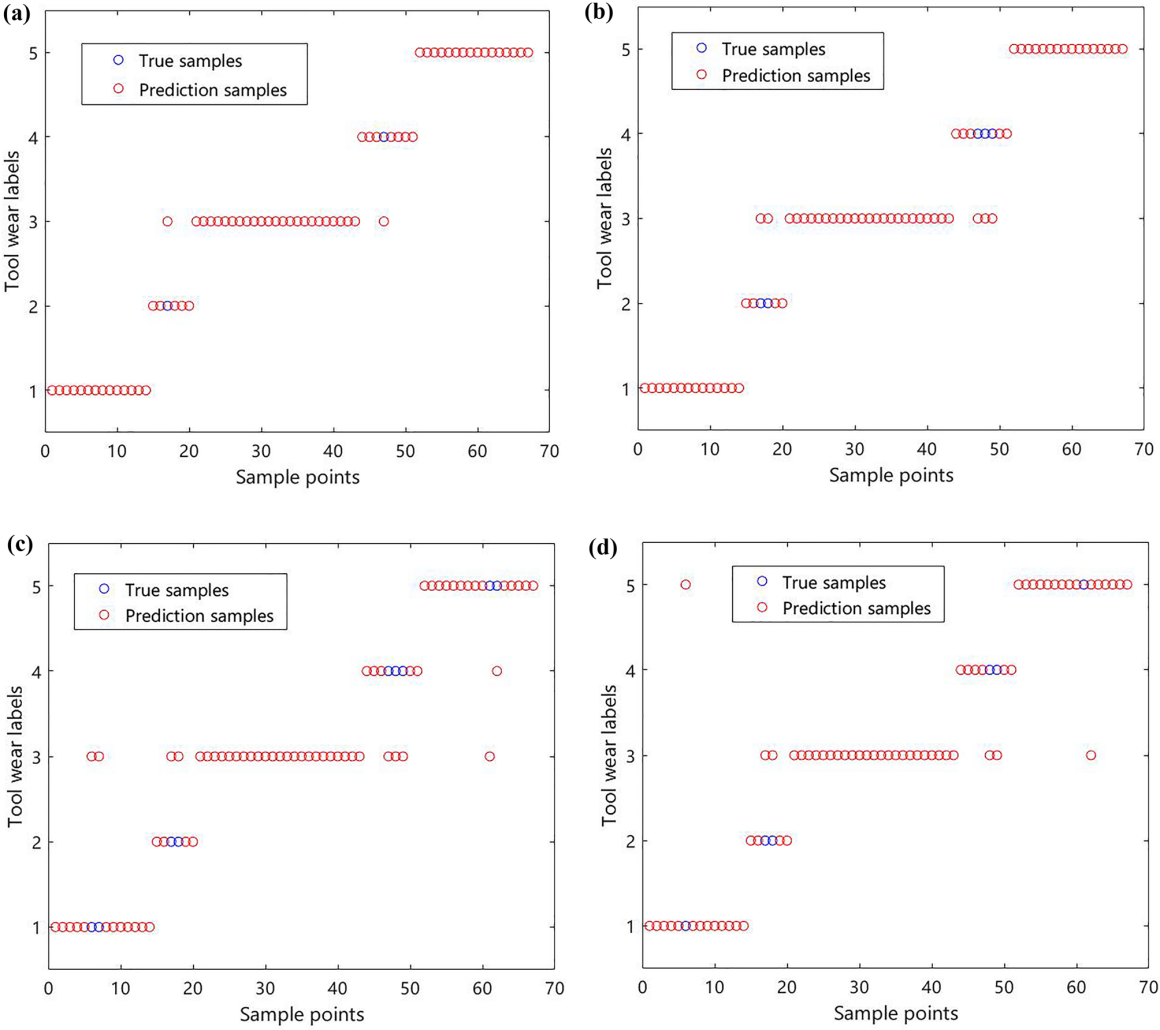
Model classification prediction results. (**a**) XGBoost (**b**) GBDT (**c**) RF (**d**) Stackincg.

From each model f1 value, there is the XGBoost model with the highest f1 value and accuracy of 96.23%, which proves that the accuracy of classification of the XGBoost model based on GS is better. Comparing the model classification time, the difference between the prediction time of the XGBoost model and the prediction time of the LGBM model is small, but the performance of the XGBoost model is improved by about 39% compared to the LGBM. Therefore, the XGBoost model also has a better performance in tool wear classification. The classification prediction results of the ensemble models of RF, GBDT, XGBoost, and Stacking models are visualized and reflected in Fig. 9. As observed in Fig. 9a, on the test set the XGBoost model misclassifies only two samples with true labels 2 and 4 as 3.

To illustrate the generalizability of the XGBoost model, this paper utilizes the PHM 2010 tool wear experimental dataset and combines the method proposed in this paper to verify the generalizability of the XGBoost model in terms of model regression and classification. Comparing the RF, GBDT, LGBM, and Stacking models, the evaluation metrics of the model prediction results are shown in Table 7.

**Table7:** Evaluation metrics for model prediction results.

Prediction models	Evaluation metrics of regression model			Evaluation metrics of classification model		
	RMSE	$R^{2}$	Prediction time	Accuracy	f1 values	Prediction time
RF	2.1641	0.9719	0.278536	0.8718	0.8650	0.031916
GBDT	3.4040	0.8484	0.084854	0.9231	0.9125	0.010989
XGBoost	1.6217	0.9801	0.063884	0.9872	0.9741	0.001997
LGBM	2.7771	0.9172	0.091806	0.5897	0.5606	0.001994
Stacking	1.7983	0.9783	0.502213	0.9103	0.9071	0.019973

From the regression and classification model prediction results, it can be seen that XGBoost has a better performance in model prediction accuracy and prediction speed compared with other prediction models (RF, GBDT, LGBM, and Stacking model). Meanwhile, the XGBoost model still performs better in 270 sets of experimental sample data done in this paper, which shows that the XGBoost model has good generalization.

**Figure 10:**
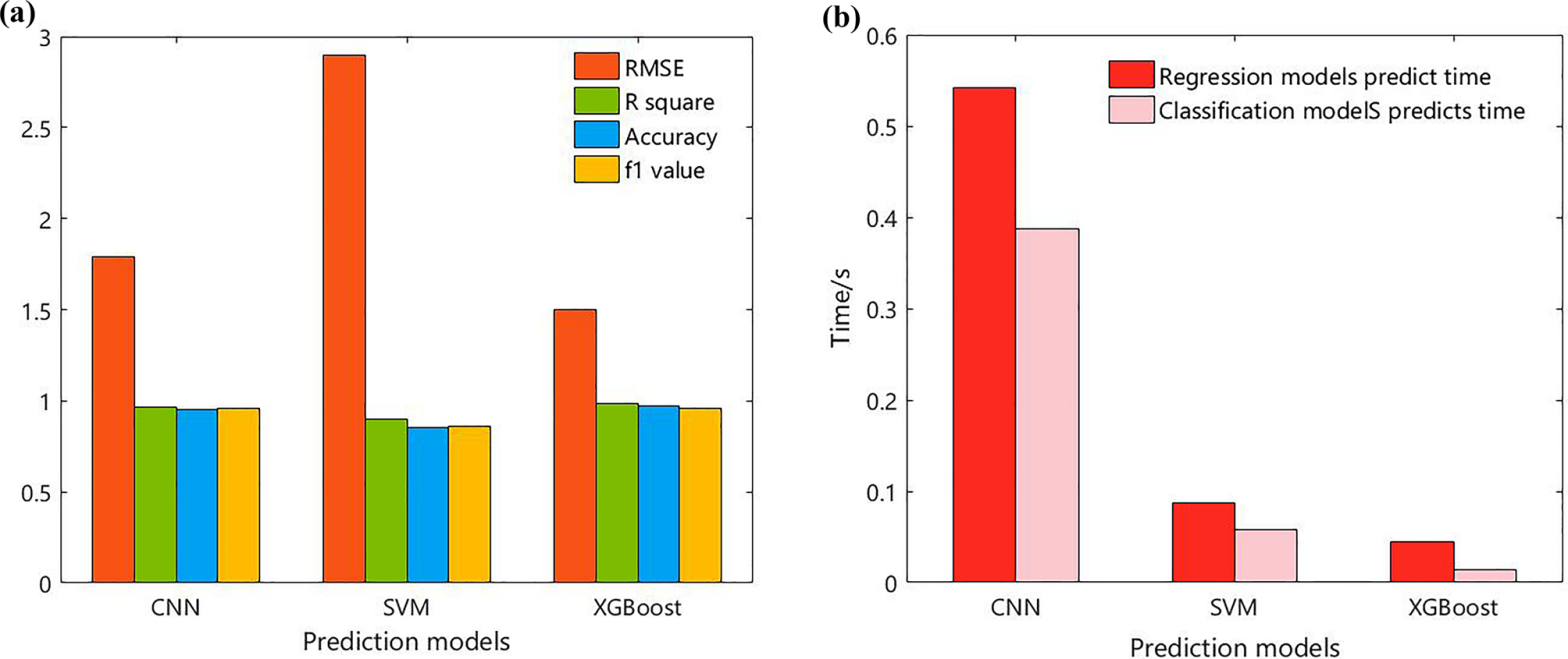
Comparative analysis of prediction performance of different models. (**a**) Models prediction accuracy (**b**) Models prediction time.

To verify the effectiveness of the DT milling cutter wear online monitoring model based on the ensemble algorithm, this paper will compare the machine learning algorithm support vector machine (SVM), and deep learning algorithm convolutional neural network (CNN). From the two aspects of the regression prediction model and classification prediction model, combined with the accuracy and prediction time evaluation index, the XGBoost ensemble model is compared and analyzed. Using the 270 sets of the experimental dataset and related settings in this paper, the model forecast results are displayed in Fig. 10. The model structure and parameters are shown below:

1. CNN model: the model consists of three convolutional layers, one dropout layer, and two fully connected layers.
2. SVM model: the regression model parameters include: the RBF function as the kernel function, the penalty coefficient $C$ = 57.8773, $\varepsilon$ = 0.014021, $\sigma$ = 0.5778; classification model parameters include: the RBF function as the kernel function for, the penalty coefficient $C$ = 46.2145, $\varepsilon$ = 0.10944, $\sigma$ = 0.4158.

As observed in Fig. 10a, in the tool wear regression and classification model prediction accuracy, XGBoost and CNN deep learning model prediction accuracy are relatively close and both are higher than 96%. The XGBoost ensemble model prediction accuracy is slightly higher than the CNN deep learning model, but both prediction accuracy is higher than the SVM machine learning model. As observed in Fig. 10b, in the tool wear regression and classification model prediction time, XGBoost and SVM machine learning model prediction time is closer and both are less than 0.1 s, and XGBoost ensemble model prediction time is slightly lower than SVM machine learning model prediction. At the same time, both model prediction times are less than the CNN deep learning model, and compared to the CNN deep learning model prediction time XGBoost ensemble model prediction time is reduced by nearly an order of magnitude. Therefore, online monitoring of milling cutter wear based on the ensemble algorithm can better predict the milling cutter wear.

## Conclusion

A digital twin-driven ensemble learning milling cutter wear online monitoring method is presented. Firstly, the DT model of the CNC machine tool is built, and the feature signals of the cutting tool machining process are gathered through sensors in real-time, and the information on the milling tool machining parameters is considered. Then, combined with the DT data multi-level processing system, the collected feature data are selected and downscaled. Finally, the ensemble model is used to predict the milling cutter wear state and wear value, which is reflected in the DT system in real-time, providing a powerful reference for tool change decisions and tool compensation strategy. The primary innovations of this paper are below:

1. The DT system for online monitoring of milling cutter wear on CNC machine tools is constructed to monitor the milling cutter situation online in real-time and analyze the milling cutter wear data visually. The predicted milling cutter wear value and wear state are mutually verified to increase the accuracy of the DT system milling cutter wear condition identification.
2. A multi-level processing system of DT milling cutter wear data is built to process the collected milling cutter wear signals online using the real-time nature of the DT. The multi-level data processing method of pre-processing, feature extraction, feature screening, and fusion optimization is adopted to ensure the integrity of feature information while minimizing feature redundancy, effectively decreasing the prediction model's calculation burden, and enhancing the speed of online monitoring of milling cutter wear by DT.
3. The ensemble algorithm is utilized to predict milling cutter wear online, which is better than other algorithms in both models predicting time and accuracy as shown by comparative analysis. The ensemble algorithm integrates multiple weak learners, so that the model stability is better, and provides strong support for the DT milling cutter wear monitoring system.

This paper utilizes digital twin technology to achieve tool wear state prediction and identification, but there are some deficiencies in this paper's research, meanwhile analyzing the limitations that exist in the research so as to point out the future research direction of this team, specifically as follows:

1. This paper is aimed at the prediction of digital twin milling cutter wear under single working conditions, when facing the prediction of digital twin milling cutter wear under variable working conditions (change of milling parameter) may not be very mature, then the team will carry out in-depth research on the digital twin cutter wear under multi-case working condition.
2. The research in this paper is carried out in the laboratory environment, involving less machining materials and the laboratory environment is more ideal, and the method proposed in this paper will have some problems in the actual operation of the factory environment. In the future, we will carry out research on digital twin tool wear monitoring for practical applications in factories.

## Data Availability

All data generated or analyzed during this study are included in this article.
