# Author Correction: An online monitoring method of milling cutter wear condition driven by digital twin

**DOI:** 10.1038/s41598-025-89670-1

**Published:** 2025-02-13

**Authors:** Xintian Zi, Shangshang Gao, Yang Xie

**Affiliations:** 1 School of Mechanical Engineering, Jiangsu University of Science and Technology, Zhenjiang, 212000 China; 2 Taian Haina Spindle Science & Technology Co., Ltd, Taian, 271000 China

Correction to: *Scientific Reports* 10.1038/s41598-024-55551-2, published online 29 February 2024

The original version of this Article omitted an affiliation for Xintian Zi. The correct affiliations are listed below.

School of Mechanical Engineering, Jiangsu University of Science and Technology, Zhenjiang, 212000, China

Taian Haina Spindle Science & Technology Co., Ltd, Taian, 271000, China

The original Article has been corrected.

